# Development of Emotion Word Comprehension in Chinese Children from 2 to 13 Years Old: Relationships with Valence and Empathy

**DOI:** 10.1371/journal.pone.0143712

**Published:** 2015-12-08

**Authors:** Yanwei Li, Dongchuan Yu

**Affiliations:** 1 Key Laboratory of Child Development and Learning Science of Ministry of Education, Southeast University, Nanjing, Jiangsu, China; 2 Research Center for Learning Science, Southeast University, Nanjing, Jiangsu, China; University of Akron, UNITED STATES

## Abstract

Children’s emotion word comprehension (EWC) has constantly received a great deal of attention in developmental science. However, since previous reports focused on only English emotion vocabulary, researchers thus far remained unclear as to the developmental trajectories of EWC (to Chinese emotion words) in Chinese children, let alone the cross-cultural difference of EWC in different languages (i.e., English V.S. Chinese). Furthermore, the influence of valence on EWC, as well as the interaction mechanism between EWC and empathy, has not been fully investigated. Finding answers to these research gaps has become the main motivation of the current study. For this purpose, a Chinese emotion vocabulary was first constructed to estimate EWC of Chinese children (ages 2–13 years old). Then, the valence of each emotion word was evaluated using the standard 9-point scale approach. After that, the Chinese children’s EWC and empathy were measured in terms of parental ratings. Finally, all data collected were statistically analyzed to reveal the influence of the valence of EWC, the relation between EWC and empathy, and the cross-cultural difference of children’s EWC between China and UK from the viewpoint of developmental science. The main results of the current study included the following: (i) EWC in general increased with age for Chinese children ages 2–13 years old, however, there was a dramatic increase during ages 6–8 years old; (ii) EWC of positive emotion words in general developed better than that of negative and neutral ones for Chinese children (ages 2–13 years old); and the disadvantage of EWC to negative emotion words over neutral ones was gradually observed with the increase of age, even though there were no significant differences between them from the beginning; (iii) EWC completely mediated the effect of age on empathy; and (iv) EWC of children in UK developed better than Chinese counterparts during the early childhood period (ages 4–6 years old), then Chinese counterparts developed better during the middle childhood period (ages 7–10 years old), however, there was no significant difference of EWC between both groups during the late childhood period (ages 11–12 years old).

## Introduction

Emotion words were regarded to be essential in describing one’s own understanding of emotion experience [1], recognizing the emotion of faces [2] or voice [3], and identifying the mental states of targeted features in movie clips [4]. So children’s emotion word comprehension (EWC) has always received a great deal of attention in developmental science [5–9]. Scientists constantly expected to understand the developmental changes of EWC in children from the very beginning. Some of them basically focused on basic emotion words (such as *happy*, *sad*, *angry*, *afraid*, *surprised*, and *disgusted*) [10–12], but others insightfully considered a broader range of emotion words (including basic and complex terms) [5, 8, 9]. For instance, Ridgeway and Waters used a list of 125 emotion words and tried to reveal the developmental changes in children aged 1.5–6 years old [9]. Baron-Cohen *et al*. suggested a much broader range of emotion words (*n* = 336) to understand the developmental trajectories of EWC in children and adolescents aged 4–16 years old [5]. Additionally, they insightfully divided these 336 emotion words into 24 emotion categories and showed interestingly that EWC of different emotion category may generally have different development trajectories and different sensitive periods. However, previous reports focused on only English emotion vocabulary. In view of this, it is of interest to reveal the developmental trajectories of EWC (to Chinese emotion words) in Chinese children, as well as the cross-cultural difference of EWC in different languages (i.e., English V.S. Chinese). Additionally, valence of stimuli may have an effect on individual’s emotion recognition [13], emotional experiences [14], emotion regulation [15, 16], and cognitive processing [17, 18], however, thus far, it still remains unknown if valence of emotion words may have an influence on EWC.

The research on empathy has attracted a large and growing amount of attention over the last two decades. There were some disagreements regarding to the operational definition of empathy, however, scientists (see [19] for a brief review) gradually agreed that empathy consists of three related but quite distinct aspects, including experience sharing, mentalizing and prosocial concern. Defects in both cognitive and affective empathy have been found in a variety of disorders, including autism spectrum disorders [20–22], schizophrenia [23, 24], bipolar disorder [23], obsessive-compulsive disorder [25], and so on. The relationships between empathy and social cognitive components were complex. Some researchers recently provided new insights into the understanding of relations among emotion comprehension, theory of mind (ToM), and empathy, and found interestingly that enhancing children’s emotion understanding may facilitate both the development of ToM and the cognitive dimension of empathy. Additionally, previous researches significantly showed that empathy did not mediate the effect of age on children’s understanding of emotions [1]. Therefore, based on observation above, it is of interest to detect whether EWC could mediate the effect of age on empathy.

Finding answers to the aforementioned questions has become the main motivation of the current study. In particular, the current research focused on revealing the developmental features of EWC in Chinese children, uncovering the influence of valence on EWC, and detecting the interaction mechanism between empathy and EWC. For those purposes, a Chinese emotion vocabulary was first constructed to estimate EWC of Chinese children (ages 2–13 years old). Then, the valence of each emotion word was evaluated using the standard 9-point scale approach [26, 27]. After that, the Chinese children’s EWC and empathy were measured in terms of parental ratings. Finally, all data collected were statistically analyzed to reveal the influence of the valence of EWC, detect the relation between EWC and empathy by a mediation analysis method [28], and discuss the cross-cultural difference of children’s EWC between China and UK to detect which culture would do better on EWC at which age groups.

## Materials and Methods

### Ethics statement

The current research was approved by the Ethics Committee of the Southeast University (located in Nanjing, Jiangsu, China). All subjects or their guardians were requested to sign the informed consent form to confirm that they knew the whole experimental procedure and agreed to have their data analyzed for scientific purposes. It should be noted that all data collected were analyzed and reported anonymously.

### Participants

188 native Chinese speakers (ages 2–13 years old) were recruited randomly from four public schools (one kindergarten and 3 elementary schools) in Nanjing, China, and their primary parents were requested to complete a series of surveys below. It should be remarked that families of participants had similar demographics such as education and socio-economic backgrounds. In addition, participants’ parents were asked to answer if their child has ever been diagnosed with any psychiatric or neurologic disorder. In this way, it was further confirmed that none of the subjects had a history of psychiatry or neurology disorder. All participants were classified into 9 age groups (2–3, 4–5, 6, 7, 8, 9, 10, 11, and 12–13 years old). 17 subjects were excluded according to our exclusion criteria cited below. The total sample was thereby *n* = 171 (85 boys) with approximately equal numbers of boys and girls at each age group. There were *n* = 16 age 2–3 years old, *n* = 19 age 4–5 years old, *n* = 24 age 6 years old, *n* = 21 age 7 years old, *n* = 20 age 8 years old, *n* = 18 age 9 years old, *n* = 17 age 10 years old, *n* = 16 age 11 years old, and *n* = 20 age 12–13 years old.

### Construction of Chinese emotion vocabulary (CEV)

The CEV basically stemmed from the translation of Baron-Cohen’s English Emotion Vocabulary (EEV) contained in a software called *Mind Reading* [5] that was suggested to improve the emotional comprehension. Two professors of English (native Chinese speakers) and two professors (native Chinese speakers but English proficient) of development psychology were first requested to translate independently Baron-Cohen’s EEV (including 412 emotion words) into Chinese. A translation-checking team with 19 persons (including 2 professors of development psychology, 10 master and 7 PhD students majoring in development psychology) then discussed the translation results and determined the final list of the CEV. It should be remarked that backward translation was not used in the current study because it was very difficult to keep the translation equivalence (see previous reports [29, 30] and discussion below for detailed information). The translation-checking team first found that Chinese counterparts of 142 English emotion words were completely consistent between translators and, more significantly, with the authorized Chinese Dictionaries. Those 142 Chinese words were thereby included in the CEV. The translation-checking team further verified that Chinese counterparts of 221 English emotion words were inconsistent between translators but each of them had similar Chinese meanings. For each of those 221 words, the translation-checking team discussed carefully the difference between translations of emotion words and finally chose the most appropriate translation according to four authorized Chinese dictionaries (i.e., *The Common Use Dictionary of Chinese*, *The Contemporary Chinese Dictionar*y, *The Modern Chinese Dictionary*, and *The Practical Chinese dictionary*) and three English-Chinese-bilingual Dictionaries (i.e., *Oxford English-Chinese Dictionary*, *Collins Learners’ English-Chinese Dictionary*, and *Longman Active Study English-Chinese Dictionary*). In this way, those 221 words were then added in the CEV. Due to the language difference, the remaining 49 emotion words were discarded because their translations were difficult to be distinguished from the confirmed emotion word list or could not be understood by the translation-checking team. Based on the steps above, the CEV with 363 words was finally constructed. Two PhD students (native Chinese speakers but English proficient) were further asked to estimate independently whether the English-to-Chinese translation for the CEV is accurate or not (see **S1 Data**). Table 1 summarizes our results and shows that the kappa coefficient of agreement is 0.6218, indicating acceptable inter-rater agreement. It should be remarked that the CEV could be further classified into 24 categories according to the work of Baron-Cohen *et al*.[5]. Table 2 listed the total word number of each emotion category in the CEV.

**Table 1:** The accuracy of the English-to-Chinese translation for the Chinese emotion vocabulary being measured with a Kappa agreement coefficient of 0.6218 between two referees, indicating acceptable inter-rater agreement.

		Referee No. 2	
		Y	N
**Referee No. 1**	**Y**	305	10
	**N**	19	29

**Table 2:** Emotion categories and the total number of words in each category in Chinese emotion vocabulary.

Group	Category	Number	Group	Category	Number
**1**	Afraid	20	**13**	Liked	8
**2**	Angry	20	**14**	Romantic	10
**3**	Bored	16	**15**	Sad	30
**4**	Bothered	7	**16**	Sneaky	10
**5**	Disbelieving	8	**17**	Sorry	7
**6**	Disgusted	3	**18**	Sure	19
**7**	Excited	15	**19**	Surprised	8
**8**	Fond	11	**20**	Thinking	8
**9**	Happy	30	**21**	Touched	4
**10**	Hurt	28	**22**	Unfriendly	45
**11**	Interested	14	**23**	Unsure	20
**12**	Kind	15	**24**	Wanting	7

### Evaluation of EWC using CEV survey

In the current study, a survey approach was used to estimate EWC of Chinese children aged 2–13 years old. For every word listed in the CEV, parents of each child participant were requested to evaluate whether their child *clearly understood*, *possibly understood* or *did not understand* its meaning. For each child participant, the total number of *clearly understood* items in parent-report was calculated as the score to evaluate EWC. A series of statistical tests were then conducted to analyze the change of EWC with the increase of age. Additionally, in order to control the quality of vocabulary survey, the final vocabulary checklist (see **S1 Table**) actually contained 382 words including all words in the CEV (*n* = 363) and 19 words being selected randomly from the CEV as the lie-detecting items. This implies that those lie-detecting items (*n* = 19) would be evaluated twice. Those parent-reports with more than 7 lie-detecting items being evaluated inconsistently (i.e., evaluation agreement less than 12/19) were excluded in the current study. Based on this standard, 17 subjects were excluded in the current study.

### Consistency of EWC evaluation between parent- and child self-report

A pilot study was conducted to detect if there was a consistency between EWC measured by parent-report and child’s self-report. For this purpose, 10 children (ages 12–13 years old, 6 boys) were recruited. Then, they and their parents were asked to complete the CEV survey independently. In this way, two EWC measures (i.e., self-report and parent-report were obtained to evaluate each child’s EWC separately. Finally, the correlation between both EWC measures was calculated. If the correlation between both EWC measures was high enough, then it was reasonable to predict that the EWC measure using parental ratings might be valid.

### Valence evaluation of emotion words

For each word in the CEV, we applied the standard valence evaluation method [26, 27] and asked 55 undergraduate and graduate students (28 males; mean age 22±2.6 years) to estimate its valence by 9-point scale starting from 1 point representing *Very Unpleasant* to 9 points representing *Very Pleasant*. By calculating statistically the scale scores of 55 participants (see **S2 Data**), the CEV can be divided into three groups, i.e., *negative emotion group* containing those words with score lower than 3, *positive emotion group* containing those words with score higher than 6, and *neutral emotion group* containing those words remaining. Based on this criterion, it was found that *negative emotion group*, *positive emotion group*, and *neutral emotion group* included 243, 101 and 19 words, respectively.

### Empathy evaluation

The Chinese version (translated by Meng-Chuan Lai & Chien-Gwo Chang, Department of Psychology, National Taiwan University) of Baron-Cohen’s Children’s version of Empathy Quotient (EQ-C) [20] was adopted to evaluate the empathy of each child participant. It should be noted that the Baron-Cohen’s EQ-C included 27 statements associated with children’s behavior in the daily life. For each statement, parents of each child participant were asked to choose the most appropriate response opinion from the four alternatives: *Definitely Agree*, *Slightly Agree*, *Slightly Disagree*, and *Definitely Disagree*, with each alternative being assigned a score according to the work of Baron-Cohen *et al*. [20]. The total score of all items (*n* = 27) was calculated as the measure of empathy.

### Mediation analysis method

In order to uncover the relationships among age, EWC and empathy, a mediation model [28] was used to identify and explicate the mechanism that underlies an observed relationship between age and empathy via the inclusion of EWC. In particular, the bootstrapping procedures provided by Preacher and Hayes [28] were conducted to detect if the presumed mediation model above is correct. To test mediation, one in general should estimate the three following regression equations: i) regressing the mediator on the independent variable; ii) regressing the dependent variable on the independent variable; and iii) regressing the dependent variable on both the independent variable and on the mediator. If the hypothesis is incorrect, the results from the mediation analysis are likely of little value. Generally, there are two kinds of mediation variables, i.e., complete and partial ones. Complete mediation is the case in which independent variable no longer affects dependent variable after mediator variable has been controlled and so direct effect path (from independent variable to dependent variable) is zero. Partial mediation is the case in which the effect of independent variable on dependent variable is reduced but still exists when the mediator is introduced.

## Results

### Development of EWC

**Fig 1:**
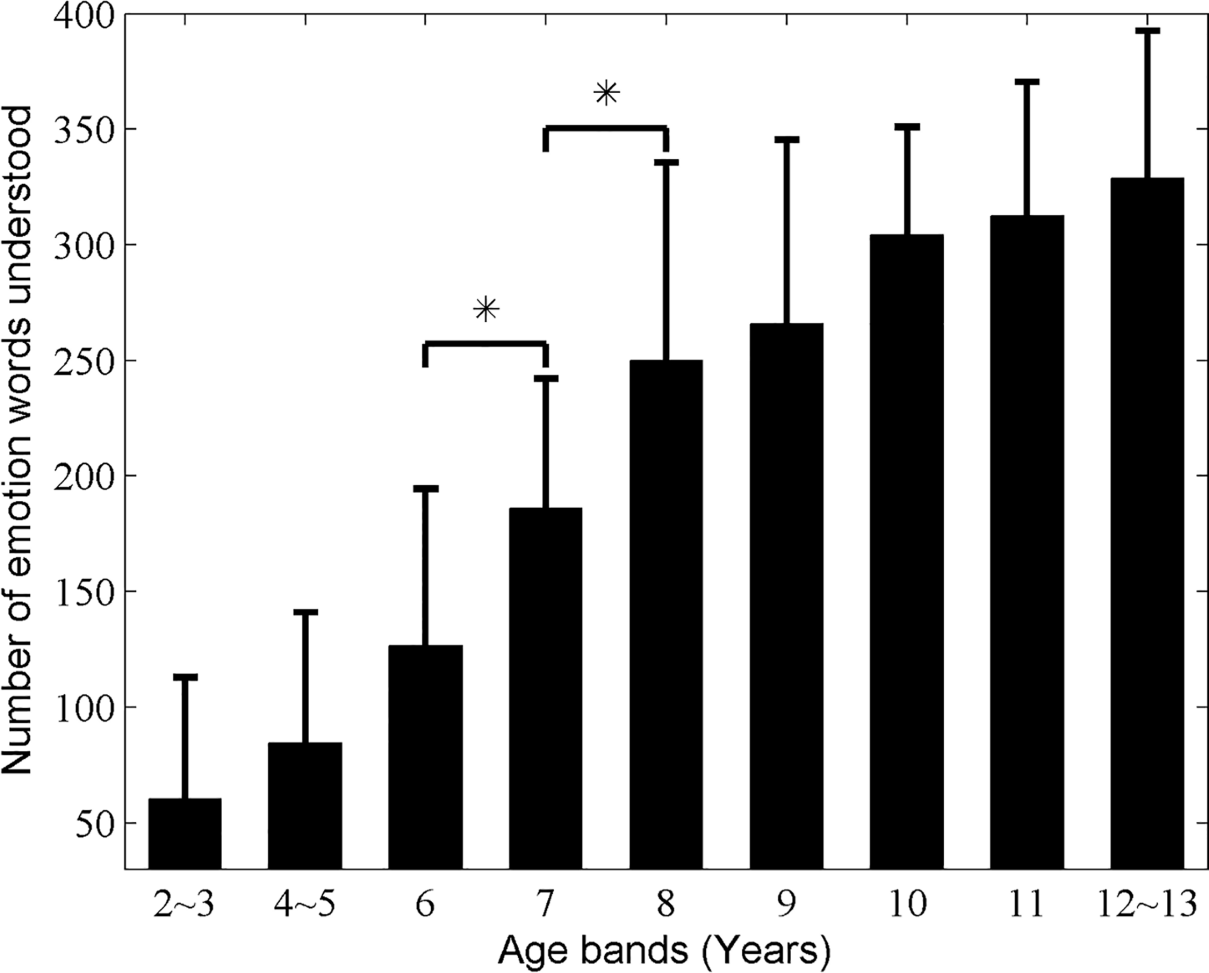
Number of emotion words understood by participants. The change of the number of emotion words understood by participants was shown with the increase of age for Chinese children aged 2–13 years old, where error bars correspond to the standard deviation and * denotes the significant difference of emotion words understanding between two neighboring age bands (p<0.0063).

All child participants’ EWC were first estimated individually by the survey (see *Material and Methods*), and between-group differences were then analyzed statistically. Fig 1 summarizes our results and illustrates the change of EWC with the increase of age for Chinese children aged 2–13 years old (see **S3 Data**). The first finding was that main effect of age on EWC was significant and EWC increased with age based on one-way ANOVA test result (***F*** = 45.643, ***p***<0.001). By a Bonferroni post hoc multiple comparison between two neighboring age groups, it was further confirmed that: (i) EWC in 7-year-olds was higher than in 6-years-olds (*p* = 0.0014, one tailed *t*-test with Bonferroni correction); and (ii) EWC in 8-year-olds was higher than in 7-year-olds (*p* = 0.0035, one tailed *t*-test with Bonferroni correction). However, there was no significant difference in other pair-comparisons with Bonferroni correction (***p***>0.0063). Those results suggested that age band 6–8 years old might be a sensitive development period for Chinese children’s EWC.

It is clear from Table 2 that the CEV was classified into 24 emotion categories. We used the percentage of emotion words understood in each emotion category to evaluate the EWC of each emotion category. Table 3 summarizes our results and shows the development of EWC in each emotion word category for Chinese children ages 2–13 years old. Our results indicated that the main effect of age was significant and EWC increased with age for all emotion word categories (*p*<0.001). By a Bonferroni post hoc multiple comparison between two neighboring age groups, it was further verified that: (i) EWC of category “touched” in age group 4–5 years old was higher than that in age group 2–3 years old (***p***<0.0045, one tailed *t*-test with Bonferroni correction); (ii) EWC of categories “disbelieving”, “happy”, and “sneaky” in age group 6-year-olds was higher than that in age group 4–5 years old (***p***<0.0052, one tailed *t*-test with Bonferroni correction); (iii) EWC of categories “afraid”, “angry”, “excited”, “fond”, “happy”, “hurt”, “interested”, “kind”, “sad”, “thinking”, “unfriendly”, “unsure”, and “wanting” in age group 7-year-olds was higher than that in age group 6-year-olds (***p***<0.0061, one tailed *t*-test with Bonferroni correction); (iv) EWC of categories “bored”, “disbelieving”, “disgusted”, “hurt”, “interested”, “sneaky”, “surprised”, “thinking”, “touched”, “unfriendly”, and “unsure” in age group 8-year-olds was higher than that in age group 7-year-olds (***p***<0.0054, one tailed *t*-test with Bonferroni correction); and (v) EWC of category “disgusted” in age group 10-year-olds was higher than that in age group 9-year-olds (***p*** = 0.0013, one tailed *t*-test with Bonferroni correction). However, there were no significant differences in the other pair-comparisons with Bonferroni correction (***p***>0.0063). Remarkably, the number of emotion categories understood by participants in age band 6–8 years old was much higher than other age bands. Such a result supports again the finding that age band 6–8 years old might be a sensitive development period for Chinese children’s EWC, even though significant differences were observed throughout 2–10 years old for Chinese children participated in the current study.

**Table 3:** Percentage of words in each emotion category understood by participants in each age band.

Category	Age (Years)								
	2–3	4–5	6	7	8	9	10	11	12–13
Afraid	15.9%	18.7%	30.8%	49.5%	63.5%	71.1%	83.5%	84.1%	89.0%
Angry	30.9%	41.1%	49.8%	67.4%	78.8%	78.6%	89.4%	90.3%	93.3%
Bored	5.5%	8.2%	22.9%	36.3%	58.8%	66.0%	77.2%	80.5%	88.1%
Bothered	18.8%	28.6%	36.3%	54.4%	66.4%	73.8%	86.6%	86.6%	88.6%
Disbelieving	7.8%	12.5%	31.3%	48.2%	71.3%	76.4%	87.5%	85.9%	90.0%
Disgusted	12.5%	12.3%	18.1%	28.6%	61.7%	50.0%	84.3%	81.3%	88.3%
Excited	14.2%	21.4%	33.1%	48.6%	62.3%	68.9%	78.0%	83.3%	85.3%
Fond	32.4%	40.7%	53.8%	70.6%	82.7%	85.9%	92.5%	93.8%	95.9%
Happy	25.6%	33.7%	52.4%	70.6%	83.2%	87.0%	92.0%	93.3%	95.8%
Hurt	12.7%	21.6%	25.3%	41.7%	62.0%	67.9%	82.1%	84.4%	87.1%
Interested	19.2%	29.3%	38.1%	59.2%	79.6%	77.8%	90.3%	90.2%	94.3%
Kind	29.6%	37.9%	53.6%	69.5%	83.3%	85.9%	91.4%	94.2%	94.7%
Liked	24.2%	28.3%	43.2%	55.4%	70.0%	77.8%	89.0%	85.9%	91.3%
Romantic	9.4%	13.2%	24.6%	40.0%	56.5%	58.9%	68.2%	79.4%	83.5%
Sad	11.3%	15.8%	26.8%	44.4%	62.0%	67.2%	76.3%	81.3%	88.3%
Sneaky	11.3%	16.8%	32.9%	44.8%	62.5%	66.1%	74.7%	76.9%	82.5%
Sorry	20.5%	26.3%	36.9%	45.6%	69.3%	71.4%	86.6%	84.8%	93.6%
Sure	12.5%	20.8%	34.6%	50.9%	70.8%	76.9%	86.1%	87.2%	93.2%
Surprised	13.3%	17.1%	28.1%	41.1%	63.1%	65.3%	75.7%	78.1%	87.5%
Thinking	10.9%	10.5%	25.0%	52.4%	75.6%	73.6%	88.2%	86.7%	91.3%
Touched	6.3%	26.3%	33.3%	35.7%	62.5%	69.4%	76.5%	76.6%	90.0%
Unfriendly	14.7%	23.2%	30.7%	44.9%	64.4%	71.0%	82.6%	86.0%	90.4%
Unsure	10.6%	15.3%	26.7%	44.3%	65.0%	71.4%	82.1%	85.6%	90.3%
Wanting	19.6%	23.3%	40.5%	66.0%	79.3%	76.2%	89.1%	93.8%	96.4%

### Influence of valence on EWC

**Fig 2:**
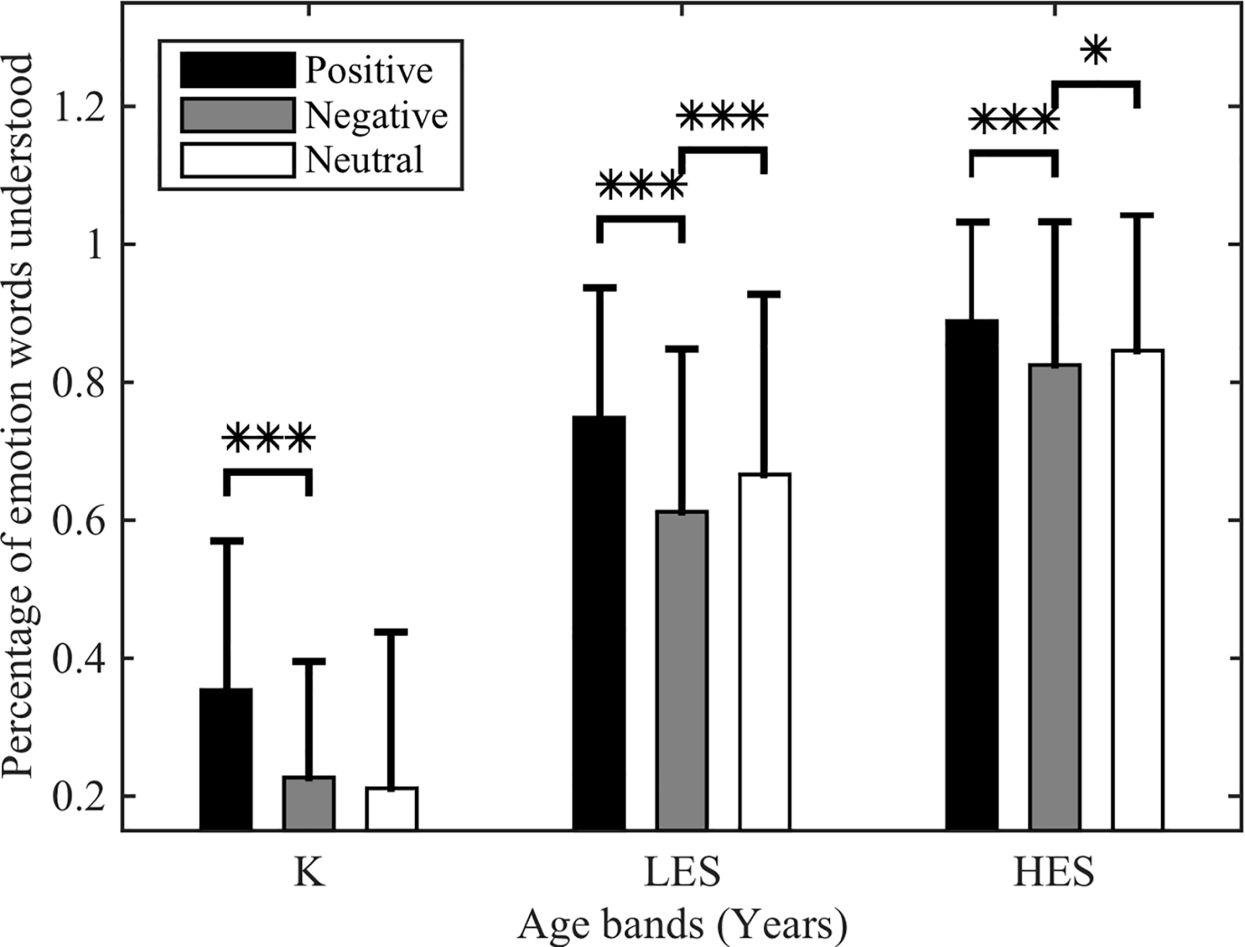
Children’s understanding of positive, negative, and neutral emotion words in each age band. Error bars correspond to the standard deviation. * and *** denote the significant difference of emotion words understanding between two neighboring age bands at the 0.05 level and 0.001 level, respectively.

As mentioned above, the CEV (n = 363) was classified into three valence groups by the standard valence evaluation method, i.e., negative emotion group (*n* = 243), positive emotion group (*n* = 101), and neutral emotion group (*n* = 19). We used the percentage of emotion words understood in a valence group to evaluate the EWC of the valence group in Chinese children aged 2–13 years old. To better investigate developmental effects of the EWC of each valence group, we followed the suggestions of work [31] and regrouped all participants into 3 age bands, i.e., kindergarten band (K-band) (ages 2–6 years old), lower elementary school band (LES-band) (ages 7–9 years old), and higher elementary school band (HES-band) (ages 10–13 years old). Fig 2 summarizes our results and shows the change of EWC of each valence group in the three age bands. A two-way 3*3 ANOVA test was adopted to confirm that the main effect of age was significant (***F*** = 130.125, ***p***<0.001). Specifically, EWC of children in HES-band was higher than that in LES-band and K-band (***p***<0.001), and EWC of children in LES-band was higher than that in K-band (***p***<0.001). Furthermore, EWC of positive emotion words was higher than that of negative or neutral ones (***p***<0.001), and EWC of neutral emotion words was higher than that of negative ones (***p*** = 0.006).

Due to the strong interaction between age and valence (***F*** = 148.578, ***p***<0.001), it was further confirmed that the EWC of positive emotion words in all age bands was higher than that of negative and neutral ones (***p***<0.001), and the EWC of neutral emotion words in HES-band and LES-band was higher than that of negative ones (***p***_***LES***_<0.001, ***p***_***HES***_ = 0.016). However, for children in K-band, there was no significance between EWC of neutral and negative emotion words (***p*** = 0.199).

### Consistency of EWC evaluation between parent- and child self-report

Since parental ratings were potentially biased, EWC evaluation using parent-report was a limitation of the current study. Therefore, it was of value to confirm the validity of parent-report. For this purpose, the current study suggested a pilot approach (see Materials and Methods) to obtain two different EWC measures by collecting and analyzing self- and parent-reported data respectively (see **S4 Data**). It was confirmed interestingly that there was a strong correlation between self- and parent-report results (r = 0.898, *p*<0.001). This indicated that the evaluation of EWC using parent-report was highly consistent with that using self-report (at least for the age 12–13 years old). This strongly supports the finding that the EWC measure using parental ratings might be valid.

### Relationship between EWC and empathy

**Fig 3:**
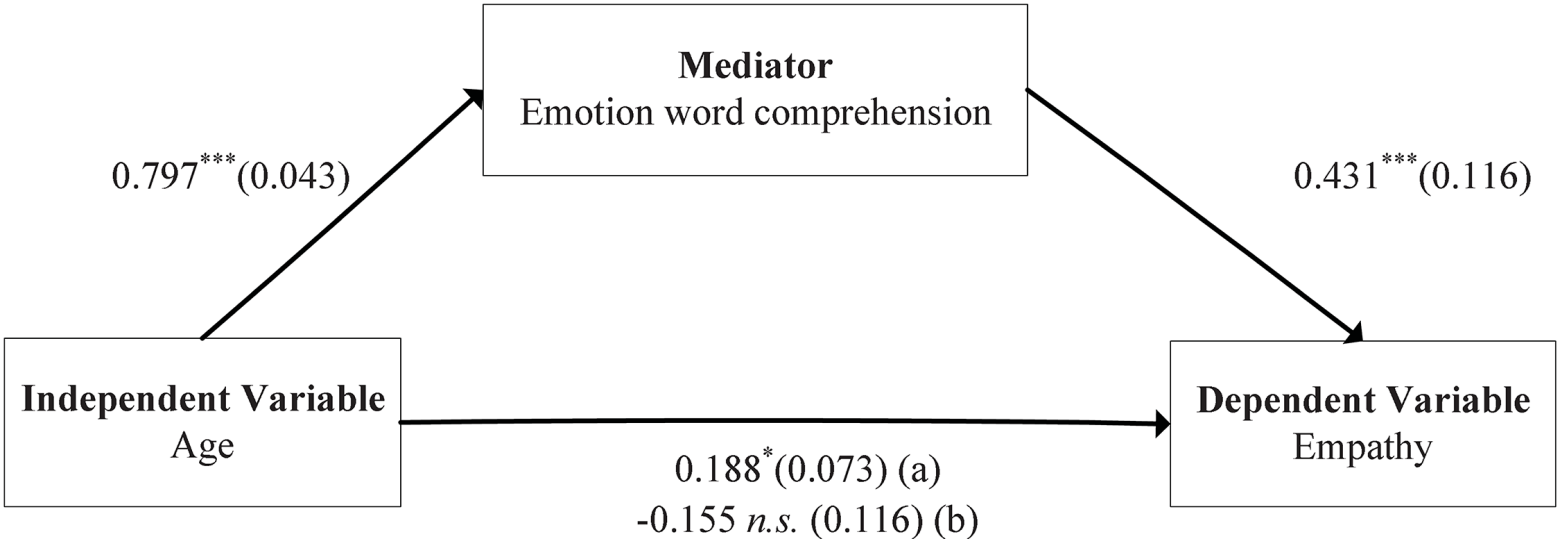
Mediation model describing the relationship among age, EWC and empathy. Path model showed Beta coefficients (Standard deviation, SD) after controlling gender in all regressions, with: (a) Beta coefficient (SD) without emotion word comprehension in the regression; and (b) Beta coefficient (SD) after controlling for emotion word comprehension in the regression. Note: *p<0.05; **p<0.01; ***p<0.001; *n*.*s*., no significance.

In terms of survey using EQ-C (see Material and Methods), all child participants’ empathy were estimated individually (see **S4 Data**). In combination with EWC measure data, it was first verified that: i) there was actually a significant positive correlation between EWC and empathy (***r*** = 0.310, ***p***<0.001); and ii) after controlling the age and gender factors, there was still a significant positive correlation between EWC and empathy (*partial* ***r*** = 0.278, ***p***<0.001). Based on this observation, the mediation model method (see Materials and Methods) with the bootstrapping procedures provided by Preacher and Hayes [28] was then applied to investigate whether EWC could mediate the effect of age on empathy. Fig 3 summarizes our results and shows the regression coefficients and their standard deviations (SDs) after controlling the effect of gender. It is clear from Fig 3 that the direct effect of age on both empathy (b = 0.188, p<0.05) and EWC (b = 0.797, ***p***<0.001) was significant for Chinese children aged 2–13 years old; when the mediator EWC inserted into the equation, the effect of age was insignificant (b = -0.155, ***p***>0.05) but the effect of EWC on empathy was significant (b = 0.431, ***p***<0.001). This indicates that EWC could be considered as a complete mediator to predict empathy for Chinese children aged 2–13 years old.

### Cross-culture comparison

As shown above, the CEV constructed was a Chinese version of Baron-Cohen’s EEV that has been insightfully applied to reveal the development of EWC in populations aged 4–16 years old in UK [5]. Therefore, it was of interest to compare the cross-cultural developmental difference of EWC in children. For this purpose, we first regrouped the data of the CEV survey and conducted statistical analysis according to the work [5]. Tables 4 and 5 summarized our results and showed the change of EWC with the increase of age.

**Table 4:** Percentage of words in each emotion category understood by at least 75% of all children in each age band.

Category	Age (Years)			
	4–6	7–8	9–10	11–12
Fond	27.27%	63.64%	90.91%	100.00%
Angry	20.00%	50.00%	85.00%	90.00%
Happy	16.67%	60.00%	86.67%	100.00%
Sorry	14.29%	14.29%	57.14%	100.00%
Kind	13.33%	53.33%	86.67%	100.00%
Liked	12.50%	37.50%	75.00%	75.00%
Sneaky	10.00%	40.00%	60.00%	50.00%
Afraid	10.00%	25.00%	65.00%	90.00%
Interested	7.14%	57.14%	85.71%	100.00%
Sure	5.26%	31.58%	73.68%	94.74%
Unfriendly	4.44%	24.44%	53.33%	82.22%
Hurt	3.57%	17.86%	57.14%	85.71%
Sad	3.33%	20.00%	46.67%	76.67%
Wanting	0.00%	57.14%	85.71%	100.00%
Bothered	0.00%	42.86%	57.14%	85.71%
Unsure	0.00%	30.00%	65.00%	80.00%
Excited	0.00%	26.67%	53.33%	80.00%
Thinking	0.00%	25.00%	75.00%	87.50%
Disbelieving	0.00%	25.00%	75.00%	87.50%
Surprised	0.00%	25.00%	62.50%	75.00%
Touched	0.00%	25.00%	25.00%	75.00%
Romantic	0.00%	20.00%	40.00%	70.00%
Bored	0.00%	18.75%	43.75%	68.75%
Disgusted	0.00%	0.00%	0.00%	66.67%

**Table 5:** Cross-cultural comparison of the development of EWC (evaluated by the number of emotion words understood). It was verified that: i) EWC of children in the UK developed better than that in the China in the early childhood period (ages 4–6 years old) but worse in the middle childhood (ages 7–10 years old); and ii) there is no significant difference between EWC of children in the UK and that in the China in the late childhood (ages 11–12 years old).

		Age (Years)			
		4–6	7–8	9–10	11–12
China	Number of participants	30	34	26	87
	Percentage of words understoodby at least 75% of participants	6.89%	33.33%	64.74%	85.40%
UK	Number of participants	42	41	35	25
	Percentage of words understoodby at least 75% of participants	12.20%	26.19%	53.57%	86.31%

Table 4 showed the percentage of words in each emotion category understood by at least 75% of participants in each age band. By a cross-cultural comparison between the current study (see Table 4) and the work [5] of UK, it was verified that different emotion categories may generally have different developmental trajectories. For instance, children aged 4–6 years old in China may understand 12.5% words in the category “liked”, but their counterparts in UK may have difficulties in understanding the meaning of emotion words in the category “liked”; children aged 4–6 years old in UK may understand 18.2% words in the category “thinking”, but their counterparts in China may show difficulties in understanding the meaning of emotion words in the category “thinking”. It was also confirmed that there was no significant between-group difference of EWC associated with category “disgusted”. Such a result is consistent with previous report [32] and seems to support that the late appearance of “disgust” might have something to do with less cultural variability.

Table 5 illustrated the cross-cultural comparison of EWC of children aged 4–11 years old in China and that in UK. Results showed by Chi-square tests were as follows: (i) EWC (evaluated by the number of emotion words understood) of children ages 4–6 years old in China was significantly lower than that in UK (*χ* ^*2*^[1] = 5.765, ***p***<0.05); (ii) EWC of children aged 7–8 years old in China was remarkably higher than that in UK (*χ* ^*2*^[1] = 4.247, ***p***<0.05); (iii) EWC of children aged 9–10 years old in China was significantly higher than that in UK (*χ* ^*2*^[1] = 9.020, ***p***<0.01); and (iv) There was no significant difference between EWC of children aged 11–12 years old in China and in UK (*χ* ^*2*^[1] = 0.119, ***p***>0.05).

## Discussion

It was of value to understand children’s EWC and its developmental trajectory due to its importance in different contexts in developmental science. However, since previous reports focused on only English emotion vocabulary, researchers thus far remained unclear as to the developmental trajectories of EWC (to Chinese emotion words) in Chinese children. Therefore, as the first aim of the current study, we expected to construct a Chinese emotion word vocabulary and then to collect normative data to document when different emotion terms are understood in development. Fig 1 and Table 3 summarized our results and showed that EWC in Chinese children aged 2–13 years old may generally increase with age and there was a dramatic increase in the age band 6–8 years old. This supported that a sensitive period for the development of EWC in Chinese children was 6–8 year olds. Additionally, EWC of different emotion category may generally have different development trajectory and different sensitive period for Chinese children. These developmental features would be helpful to assess emotion-comprehension delays or deficits for clinical purposes in terms of EWC with respect to his/her reference population.

Valence of stimuli may influence not only emotional performance but also cognitive processing, including working memory [33], cognitive control [34], language [35], and decision making [17, 18]. However, it remained poorly understood if EWC to an emotion word could be affected by its valence. Therefore, the second aim of the current study was to investigate the influence of valence on EWC. Fig 2 summarized our results and showed that children may generally have different EWC to emotion words with different valence. More precisely, it was confirmed that the EWC of positive emotion category in general developed better than of negative and neutral categories in Chinese children aged 2–13 years old. In addition, the disadvantage of EWC to negative emotion words over neutral ones was gradually observed with the increase of age, even though there was no significant difference between them from the very beginning. Therefore, our results seemed to support the development of EWC might have the inclination of loving the positive but alienating the negative for Chinese children. This advantage of positive emotion over negative emotion might be partially interpreted and supported by high operation performance for positive emotion. Typical results, for instance, included the following: i) pre-school children had a higher naming accuracy for positive facial expressions than negative ones [36], and ii) adults had a higher recognition accuracy for positive facial expressions than negative ones [37]. Additionally, as shown above, *negative emotion group* and *positive emotion group* included 243 and 101 words, respectively. This indicates that the emotion lexicon for negative emotion is much more elaborated and specific than for positive emotion and thereby children might have a higher risk to make mistakes during learning the knowledge of negative emotions [38, 39].

The research on empathy has attracted a large and growing amount of attention over the last two decades. It was considered as a major part of “emotional intelligence” [40] and recently was closely connected to the neurobiological discovery of “mirror neurons” [41]. However, the relationships among age, empathy and cognitive components were complex, and still remained poorly understood. Empathy was a multidimensional construct consisting of cognitive (inferring mental states) and emotional (empathic concern) components [19–25, 42–46]. It was clear that EWC could be considered as a fundamental component used to measure cognitive empathy, but it was still unknown whether there is a causal relationship between EWC and empathy. On the other hand, the development of empathy has been investigated [47–50]. In particular, some scientists showed interestingly that children aged 2 years old normally began to display the fundamental behaviors of empathy and to generate an emotional response corresponding with another person's emotional state [50]. Additionally, previous researches significantly showed [1] that empathy did not mediate the effect of age on children’s understanding of emotions. Based on observation above, the third aim of the current study was thereby to detect whether EWC could completely, partially, or impossibly mediate the effect of age on empathy by the mediation model method (see Materials and Methods) with the bootstrapping procedures [28]. Our results remarkably showed that EWC could be considered as a complete mediator to predict empathy. However, one still could not infer the existence of the directional relationship between EWC and empathy from the current cross-sectional data. Future longitudinal research is necessary to further examine directionality of effects for the interrelations between age, EWC and empathy.

Mediation analysis has been widely used in developmental science, since it allowed interesting associations to be decomposed into components that reveal possible causal mechanisms. The underlying mediation models were useful for theory development and testing as well as for the identification of possible points of intervention in applied work. Statistical approaches to the analysis of mediation have been discussed extensively in the psychological literature. One particularly useful approach was the bootstrapping framework [28], which was of nonparametric identification and was an additional method advocated for testing mediation that did not impose the assumption of normality of the sampling distribution, and, more significantly, could be applied even when sample sizes were moderate or small, that is, in the range of 20–80 cases [51]. Although the bootstrapping was well-known to statisticians and has been incorporated as an option in structural equation modeling programs such as AMOS, it has only recently begun to appear in the general psychology literature [28]. The current study illustrated its power and did not require any statistical assumption about the data set for the inference of mediation.

A parent-report method was suggested to estimate EWC of children (ages 2–13 years old). This was clearly a limitation of the current study because parental reports might be culture-biased and it might be more reflective of their expectations rather than the children’s actual level of capability [52]. Actually, previous researches reported that parents’ attitude toward their children’s success and failure were culture-biased and even would have an effect on children’s academic achievements [53–55]. Therefore, in such circumstance, it seemed more reliable if an actual performance evaluation associated with confirmation of EWC was conducted independently in laboratory. However, such kind of test could not be suitable for a large-scale survey of comprehension like the current study. Fortunately, as mentioned by Baron-Cohen *et al*. [5], some scientists verified that parent-report correlated well with actual performance measure of language comprehension. Additionally, the current study showed interestingly that there was a high correlation between self-reported and parent-reported data (r = 0.898, *p*<0.001). This indicated that the evaluation of EWC using parent-report was highly consistent with that using self-report (at least for children ages 12–13 years old). This strongly supported the finding that the EWC measure reported by parents might be valid. It should be noted that since all developmental variables (i.e., EWC and empathy) used in the current research were reported by parents, the potentially cultural bias might have been adjusted in the mediation analysis.

The cross-cultural comparison results seemed to support the hypothesis that: (i) For the early childhood period (age band 4–6 years old), EWC of children in UK developed better than that in China; (ii) For the middle childhood period (age band 7–10 years old), EWC of children in China developed better than that in UK; and (iii) For the late childhood period (age band 11–12 years old), there was no significant difference of EWC between children in China and in UK. The hypothesis might reasonably be interpreted by the differences of language and educational systems between both nations. However, detailed analysis is beyond the scope of the current study and requires more subjects and a more serious cross-cultural design.

Previous researches discussed carefully the translation equivalence of emotion words between different languages, and clarified that even when best translations for emotion terms were available, they often anchored distinct conceptual contents [29, 30, 56, 57] and were definitely not the equivalent pairs [29, 56]. As a result, backward translation was not used in the current study. In addition, it would be of great value to reveal the developmental features of Chinese children’s EWC by using emotion words list constructed directly from native Chinese daily-life dictionary, instead of the current version translated from English. However, in such circumstance, the cross-cultural comparison between different languages would become quite complex and difficult.

It has been widely accepted that there are only a small number of basic emotions that are recognized cross-culturally, but complex emotions usually are treated as being culture-specific. This view has been partially supported by the cross-cultural comparison between UK and China that children in UK developed better in understanding some kinds of emotions but more slowly in others during the early and middle childhood period. However, it was also verified interestingly that children in both nations showed almost the same developmental trajectories in understanding some kinds of emotions (e.g., category “disgusted” [5]). This seemed to hypothesize that some complex emotions could still be recognized cross-culturally or be less culture-specific. Further discussion deserves to be analyzed in detail in the future.

## Conclusions

Since previous reports focused on only English emotion vocabulary, this was the first time to systematically uncover the developmental trajectories of EWC (to Chinese emotion words) in Chinese children and reveal the cross-cultural difference between EWC of children in China and that in UK. In addition, the current study expected to: i) reveal the relationship between EWC and empathy, ii) detect whether valence of an emotion word had an influence on its EWC, and iii) discuss the consistency of EWC evaluation between parent- and child self-report. The main results of the current study included the following: i) a Chinese emotion vocabulary (*n* = 363) was constructed with the valence of each emotion word being evaluated; ii) EWC generally increased with age for Chinese children aged 2–13 years old, and there was a dramatic increase during 6–8 years old; iii) the development of EWC may have the inclination of loving the positive but alienating the negative for Chinese children; iv) EWC could completely mediate the effect of age on empathy for Chinese children; v) the evaluation of EWC using parental ratings might be valid because it was highly consistent with self-report data (at least for the ages 12–13 years old); and vi) EWC of children in UK developed better than Chinese counterparts during the early childhood period, then Chinese counterparts developed better during the middle childhood period, but, not surprisingly, there was no significant difference of EWC between both groups during the late childhood period. The data reported here may be used to reveal developmental features of EWC in Chinese children, detect the developmentally sensitive period for different Chinese emotion words, label emotions of Chinese experimental materials (including picture, text, voice, and movies), support cross-cultural comparison researches, and assess emotion-comprehension delays or deficits for clinical purposes in terms of emotion word comprehension with respect to his/her reference population.

## Supporting Information

S1 Data The original data of two referees used to measure the accuracy of the English-to-Chinese translation for the Chinese emotion vocabulary. (XLS)

S2 Data The orginal data used to measure valence of Chinese emotion words in the CEV. It was found that the CEV can be divided into three groups, i.e., negative emotion group containing those words with score lower than 3, positive emotion group containing those words with score higher than 6, and neutral emotion group containing those words remaining.
(SAV)

S3 Data The orginal data used to measure EWC and empathy of children participated, with EQi and Ti corresponding to the i-th question of empathy and CEV surveys, respectively. (SAV)

S4 Data The orginal parent- and self-reported data used to measure independently EWC of children participated, with ID nA and nB corresponding to parent- and self-reported data of the n-th child, respecitively. (SAV)

S1 Table The final checklist for the CEV survey used to measure EWC of Children. (DOC)

## References

[1] Zajdel RT, Bloom JM, Fireman G, Larsen JT. Children's Understanding and Experience of Mixed Emotions: The Roles of Age, Gender, and Empathy. J Genet Psychol. 2013;174(5):582–603. 10.1080/00221325.2012.732125 . 24303574

[2] Widen S, Russell J. Children's and adults' understanding of the disgust face. Cognition Emotion. 2008;22(8):1513–41. Pii 791354917.

[3] Loveland KA, Tunalikotoski B, Chen R, Brelsford KA, Ortegon J, Pearson DA. Intermodal Perception of Affect in Persons with Autism or down-Syndrome. Dev Psychopathol. 1995;7(3):409–18. .

[4] Golan O, Baron-Cohen S, Golan Y. The 'Reading the Mind in Films' task [child version]: Complex emotion and mental state recognition in children with and without autism spectrum conditions. J Autism Dev Disord. 2008;38(8):1534–41. 10.1007/s10803-007-0533-7 . 18311514

[5] Baron-Cohen S, Golan O, Wheelwright S, Granader Y, Hill J. Emotion word comprehension from 4 to 16 years old: a developmental survey. Front Evol Neurosci. 2010;2:109 Epub 2010/12/15. 10.3389/fnevo.2010.00109 21151378 PMC2996255

[6] Bretherton I, McNew S, Beeghly M. Early person knowledge in gestural and verbal communication: When do infants acquire a "theory of mind"? In Lamb M. & Sherrod L. (Eds.), Infant so Hillsdale, NJ: Erlbaum; 1981.

[7] Bretherton L, Beeghly M. Talking about internal states: The acquisition of an explicit theory of mind. Dev Psychol. 1982;19:906–21.

[8] Pochon R, Declercq C. Emotion recognition by children with Down syndrome: A longitudinal study. J Intellect Dev Dis. 2013;38(4):332–43. 10.3109/13668250.2013.826346 . 24279786

[9] Ridgeway D, Waters E, Kuczaj SA. Acquisition of emotion descriptive language: receptive and productive vocabulary norms for ages 18 months to 6 years. Developmental Psycholology. 1985;21:901–8.

[10] Ekman P, Friesen WV. Constants across cultures in the face and emotion. J Pers Soc Psychol. 1971;17(2):124–9. Epub 1971/02/01. . 5542557 10.1037/h0030377

[11] Ekman P. Facial Expression and Emotion. Am Psychol. 1993;48(4):384–92. 10.1037//0003-066x.48.4.384 . 8512154

[12] Herba C, Phillips M. Annotation: Development of facial expression recognition from childhood to adolescence: behavioural and neurological perspectives. J Child Psychol Psyc. 2004;45(7):1185–98. 10.1111/j.1469-7610.2004.00316.x . 15335339

[13] Eyben F, Wollmer M, Graves A, Schuller B, Douglas-Cowie E, Cowie R. On-line emotion recognition in a 3-D activation-valence-time continuum using acoustic and linguistic cues. J Multimodal User In. 2010;3(1–2):7–19. 10.1007/s12193-009-0032-6 .

[14] Christensen JF, Gaigg SB, Gomila A, Oke P, Calvo-Merino B. Enhancing emotional experiences to dance through music: the role of valence and arousal in the cross-modal bias. Front Hum Neurosci. 2014;8 10.3389/fnhum.2014.00757 . PMC4186320 25339880

[15] Morris JA, Leclerc CM, Kensinger EA. Your Attention Please: Effects of Valence on Emotion Regulation with Full- and Divided-Attention. J Cognitive Neurosci. 2013:182–3. .

[16] Vrticka P, Sander D, Vuilleumier P. Effects of emotion regulation strategy on brain responses to the valence and social content of visual scenes. Neuropsychologia. 2011;49(5):1067–82. 10.1016/j.neuropsychologia.2011.02.020 . 21345342

[17] Carmona-Perera M, Marti-Garcia C, Perez-Garcia M, Verdejo-Garcia A. Valence of emotions and moral decision-making: increased pleasantness to pleasant images and decreased unpleasantness to unpleasant images are associated with utilitarian choices in healthy adults (vol 7, 626, 2013). Front Hum Neurosci. 2014;8 Artn 50. 10.3389/Fnhum.2014.00050 . PMC3783947 24133433

[18] de Hooge IE, Verlegh PWJ, Tzioti SC. Emotions in Advice Taking: The Roles of Agency and Valence. J Behav Decis Making. 2014;27(3):246–58. 10.1002/Bdm.1801 .

[19] Zaki J, Ochsner KN. The neuroscience of empathy: progress, pitfalls and promise. Nat Neurosci. 2012;15(5):675–80. 10.1038/nn.3085 . 22504346

[20] Auyeung B, Wheelwright S, Allison C, Atkinson M, Samarawickrema N, Baron-Cohen S. The Children's Empathy Quotient and Systemizing Quotient: Sex Differences in Typical Development and in Autism Spectrum Conditions. J Autism Dev Disord. 2009;39(11):1509–21. 10.1007/s10803-009-0772-x . 19533317

[21] Pasalich DS, Dadds MR, Hawes DJ. Cognitive and affective empathy in children with conduct problems: Additive and interactive effects of callous-unemotional traits and autism spectrum disorders symptoms. Psychiat Res. 2014;219(3):625–30. 10.1016/j.psychres.2014.06.025 . 25015711

[22] Rueda P, Fernandez-Berrocal P, Baron-Cohen S. Dissociation between cognitive and affective empathy in youth with Asperger Syndrome. Eur J Dev Psychol. 2015;12(1):85–98. 10.1080/17405629.2014.950221 .

[23] Baez S, Herrera E, Villarin L, Theil D, Gonzalez-Gadea ML, Gomez P, et al Contextual Social Cognition Impairments in Schizophrenia and Bipolar Disorder. Plos One. 2013;8(3). ARTN e57664 10.1371/journal.pone.0057664 . PMC3592887 23520477

[24] Brune M. "Theory of mind" in schizophrenia: A review of the literature. Schizophrenia Bull. 2005;31(1):21–42. 10.1093/schbul/sbi002 . 15888423

[25] Kang JI, Namkoong K, Yoo SW, Jhung K, Kim SJ. Abnormalities of emotional awareness and perception in patients with obsessive-compulsive disorder. J Affect Disorders. 2012;141(2–3):286–93. 10.1016/j.jad.2012.04.001 . 22542863

[26] Montefinese M, Ambrosini E, Fairfield B, Mammarella N. The adaptation of the Affective Norms for English Words (ANEW) for Italian. Behav Res Methods. 2014;46(3):887–903. 10.3758/s13428-013-0405-3 . 24150921

[27] Soares AP, Comesana M, Pinheiro AP, Simoes A, Frade CS. The adaptation of the Affective Norms for English Words (ANEW) for European Portuguese. Behav Res Methods. 2012;44(1):256–69. 10.3758/s13428-011-0131-7 . 21751068

[28] Preacher KJ, Hayes AF. Asymptotic and resampling strategies for assessingand comparing indirect effects in multiple mediator models. Behav Res Methods. 2008;40(879–891). 10.3758/brm.40.3.879 18697684

[29] Russell JA. Culture and the Categorization of Emotions. Psychol Bull. 1991;110(3):426–50. 10.1037/0033-2909.110.3.426 . 1758918

[30] Wierzbicka A. Language and Metalanguage: Key Issues in Emotion Research. Emot Rev. 2009;1(1):3–14. 10.1177/1754073908097175 .

[31] Kayyal MH, Russell JA. Language and Emotion: Certain English-Arabic Translations Are Not Equivalent. J Lang Soc Psychol. 2013;32(3):261–71. 10.1177/0261927x12461004 .

[32] Widen SC, Russell JA. A closer look at preschoolers' freely produced labels for facial expressions. Dev Psychol. 2003;39(1):114 12518813 10.1037//0012-1649.39.1.114

[33] Bergmann HC, Rijpkema M, Fernandez G, Kessels RPC. The Effects of Valence and Arousal on Associative Working Memory and Long-Term Memory. Plos One. 2012;7(12). ARTN e52616. 10.1371/journal.pone.0052616 . PMC3530455 23300724

[34] Harle KM, Shenoy P, Paulus MP. The influence of emotions on cognitive control: feelings and beliefs—where do they meet? Front Hum Neurosci. 2013;7 Artn 508. 10.3389/Fnhum.2013.00508 . PMC3776943 24065901

[35] Kuperman V, Estes Z, Brysbaert M, Warriner AB. Emotion and Language: Valence and Arousal Affect Word Recognition. J Exp Psychol Gen. 2014;143(3):1065–81. 10.1037/A0035669 . 24490848 PMC4038659

[36] Denham SA. Social cognition, prosocial behavior, and emotion in preschoolers: Contextual validation. Child Dev. 1986;57:194–201.

[37] Jansari A, Rodway P, Goncalves S. Identifying facial emotions: Valence specific effects and an exploration of the effects of viewer gender. Brain Cognition. 2011;76(3):415–23. 10.1016/j.bandc.2011.03.009 . 21514027

[38] Schrauf RW, Sanchez J. The preponderance of negative emotion words in the emotion lexicon: A cross-generational and cross-linguistic study. Journal of Multilingual and Multicultural Development. 2004;25(2–3):266–84.

[39] Marian V, Kaushanskaya M. Words, feelings, and bilingualism: Cross-linguistic differences in emotionality of autobiographical memories*. The mental lexicon. 2008;3(1):72–90. 19966924 10.1075/ml.3.1.06mar PMC2788822

[40] Salovey P, Mayer JD. Emotional intelligence. Imagination, cognition and personality. 1990;9(3):185–211.

[41] Gallese V. The'shared manifold'hypothesis. From mirror neurons to empathy. Journal of consciousness studies. 2001;8(5–7):33–50.

[42] Carlo G, McGinley M, Hayes RC, Martinez MM. Empathy as a mediator of the relations between parent and peer attachment and prosocial and physically aggressive behaviors in Mexican American college students. J Soc Pers Relat. 2012;29(3):337–57. 10.1177/0265407511431181 .

[43] Lockwood PL, Seara-Cardoso A, Viding E. Emotion Regulation Moderates the Association between Empathy and Prosocial Behavior. Plos One. 2014;9(5). ARTN e96555. 10.1371/journal.pone.0096555 . PMC4014517 24810604

[44] Parkin T, de Looy A, Farrand P. Greater professional empathy leads to higher agreement about decisions made in the consultation. Patient Educ Couns. 2014;96(2):144–50. 10.1016/j.pec.2014.04.019 . 24857331

[45] Pijnenborg GHM, Spikman JM, Jeronimus BF, Aleman A. Insight in schizophrenia: associations with empathy. Eur Arch Psy Clin N. 2013;263(4):299–307. 10.1007/s00406-012-0373-0 . 23076736

[46] Topcu C, Erdur-Baker O. Affective and cognitive empathy as mediators of gender differences in cyber and traditional bullying. School Psychol Int. 2012;33(5):550–61. 10.1177/0143034312446882 .

[47] Decety J, Meyer M. From emotion resonance to empathic understanding: A social developmental neuroscience account. Dev Psychopathol. 2008;20(04):1053–80. 18838031 10.1017/S0954579408000503

[48] Eisenberg N, Spinrad TL, Sadovsky A. Empathy-related responding in children. Handbook of moral development. 2006:517–49.

[49] Falck-Ytter T, Gredebäck G, von Hofsten C. Infants predict other people's action goals. Nature neuroscience. 2006;9(7):878–9. 16783366 10.1038/nn1729

[50] Hoffman ML. Empathy and moral development: Implications for caring and justice: Cambridge University Press; 2001.

[51] Efron B, Tibshirani RJ. An Introduction to the Bootstrap, Monographs on Statistics and Applied Probability, Vol. 57 New York and London: Chapman and Hall/CRC 1993.

[52] Chen C, Uttal DH. Cultural values, parents' beliefs, and children’s achievement in the United States and China. Human Development. 1988;31(6):351–8.

[53] Hess RD, Chang C-M, McDevitt TM. Cultural variations in family beliefs about children's performance in mathematics: Comparisons among People's Republic of China, Chinese-American, and Caucasian-American families. Journal of Educational Psychology. 1987;79(2):179.

[54] Stevenson HW, Chen C, Lee S-Y. Mathematics achievement of Chinese, Japanese, and American children: Ten years later. Science. 1993; 259(5091):53–8. 8418494 10.1126/science.8418494

[55] Stevenson HW, Lee S-Y, Stigler JW. Mathematics achievement of Chinese, Japanese, and American children. Science. 1986;231(4739):693–9. 3945803 10.1126/science.3945803

[56] Kayyal MH, Russell JA. Language and emotion: certain English–Arabic translations are not equivalent. J Lang Soc Psychol. 2013:0261927X12461004.

[57] Wierzbicka A. Is pain a human universal? A cross-linguistic and cross-cultural perspective on pain. Emot Rev. 2012;4(3):307–17.

