# Supplementary material for: Development of Emotion Word Comprehension in Chinese Children from 2 to 13 Years Old: Relationships with Valence and Empathy
Source: PLoS One. 2015 Dec 8;10(12):e0143712. doi: 10.1371/journal.pone.0143712 (PMC4672887; doi:10.1371/journal.pone.0143712)
Supplement: S1 Table — (DOC) [file pone.0143712.s005.doc]

Chinese emotion words list for survey

| No. | Word |
| --- | --- |
| 1 | 哀求的 |
| 2 | 挨批评的 |
| 3 | 爱护 |
| 4 | 傲慢 |
| 5 | 霸道的 |
| 6 | 抱歉的 |
| 7 | 抱怨的 |
| 8 | 暴怒 |
| 9 | 爆躁的 |
| 10 | 卑鄙 |
| 11 | 悲苦 |
| 12 | 悲恸 |
| 13 | 被称赞的 |
| 14 | 被出卖的 |
| 15 | 信任的 |
| 16 | 被反驳的 |
| 17 | 被恭维的 |
| 18 | 被忽略的 |
| 19 | 被忽视的 |
| 20 | 被怀疑的 |
| 21 | 被激惹的 |
| 22 | 被冒犯的 |
| 23 | 被蔑视的 |
| 24 | 被迫的 |
| 25 | 被轻视的 |
| 26 | 被吸引的 |
| 27 | 鄙视的 |
| 28 | 不忿 |
| 29 | 不满的 |
| 30 | 不情愿的 |
| 31 | 不爽 |
| 32 | 不友好的 |
| 33 | 不愉快的 |
| 34 | 不知所措 |
| 35 | 猜忌的 |
| 36 | 猜疑的 |
| 37 | 残忍 |
| 38 | 谄媚的 |
| 39 | 沉思的 |
| 40 | 沉着 |
| No. | Word |
| 41 | 诚实 |
| 42 | 斥责的 |
| 43 | 炽情的 |
| 44 | 仇视的 |
| 45 | 出神的 |
| 46 | 欢快 |
| 47 | 触动的 |
| 48 | 粗暴 |
| 49 | 粗鲁 |
| 50 | 脆弱 |
| 51 | 挫败的 |
| 52 | 呆滞 |
| 53 | 担心的 |
| 54 | 担忧的 |
| 55 | 胆怯 |
| 56 | 淡漠 |
| 57 | 敌意的 |
| 58 | 抵触的 |
| 59 | 惦念的 |
| 60 | 恫吓的 |
| 61 | 抖擞的 |
| 62 | 对质的 |
| 63 | 多愁善感 |
| 64 | 恶心 |
| 65 | 愕然 |
| 66 | 发牢骚的 |
| 67 | 发怒的 |
| 68 | 烦恼的 |
| 69 | 烦扰 |
| 70 | 被激惹的 |
| 71 | 烦躁 |
| 72 | 反对的 |
| 73 | 反感的 |
| 74 | 放松的 |
| 75 | 愤愤不平 |
| 76 | 愤慨 |
| 77 | 愤懑 |
| 78 | 愤怒 |
| 79 | 奉承的 |
| 80 | 尴尬 |
| No. | Word |
| 81 | 感动的 |
| 82 | 感激的 |
| 83 | 感兴趣的 |
| 84 | 高兴 |
| 85 | 恭敬 |
| 86 | 孤傲 |
| 87 | 孤独 |
| 88 | 蛊惑的 |
| 89 | 鼓励的 |
| 90 | 固执 |
| 91 | 关切的 |
| 92 | 亲昵 |
| 93 | 关心的 |
| 94 | 鬼鬼祟祟 |
| 95 | 果断 |
| 96 | 害怕的 |
| 97 | 害羞 |
| 98 | 好辩的 |
| 99 | 好奇 |
| 100 | 合群 |
| 101 | 厚颜无耻 |
| 102 | 糊涂 |
| 103 | 怀疑的 |
| 104 | 欢快 |
| 105 | 欢欣鼓舞 |
| 106 | 幻灭的 |
| 107 | 幻想的 |
| 108 | 唤醒的 |
| 109 | 涣散 |
| 110 | 慌乱 |
| 111 | 恍惚 |
| 112 | 无礼的 |
| 113 | 回绝的 |
| 114 | 活泼的 |
| 115 | 讥讽的 |
| 116 | 机警 |
| 117 | 积极的 |
| 118 | 激情四射 |
| 119 | 嫉妒的 |
| 120 | 记恨的 |
| No. | Word |
| 121 | 坚决 |
| 122 | 坚强 |
| 123 | 煎熬的 |
| 124 | 焦虑 |
| 125 | 搅扰的 |
| 126 | 紧张 |
| 127 | 谨慎的 |
| 128 | 惊愕的 |
| 129 | 惊骇的 |
| 130 | 疲倦 |
| 131 | 惊慌失措 |
| 132 | 惊恐 |
| 133 | 惊叹的 |
| 134 | 惊喜的 |
| 135 | 惊吓的 |
| 136 | 精力充沛的 |
| 137 | 警觉的 |
| 138 | 警惕的 |
| 139 | 敬畏的 |
| 140 | 沮丧 |
| 141 | 决心的 |
| 142 | 绝望的 |
| 143 | 开心 |
| 144 | 慷慨的 |
| 145 | 亢奋的 |
| 146 | 渴望的 |
| 147 | 渴望的 |
| 148 | 恳求的 |
| 149 | 空虚 |
| 150 | 苦恼的 |
| 151 | 贪婪的 |
| 152 | 快乐 |
| 153 | 宽慰 |
| 154 | 狂怒的 |
| 155 | 狂躁 |
| 156 | 愧疚的 |
| 157 | 困惑 |
| 158 | 困扰的 |
| 159 | 浪漫 |
| 160 | 泪汪汪的 |
| 161 | 冷淡 |
| 162 | 冷酷 |
| No. | Word |
| 163 | 冷落的 |
| 164 | 冷漠 |
| 165 | 理解的 |
| 166 | 怜爱的 |
| 167 | 怜悯的 |
| 168 | 领悟的 |
| 169 | 六神无主 |
| 170 | 卖俏的 |
| 171 | 满足的 |
| 172 | 漫不经心 |
| 173 | 茫然的 |
| 174 | 矛盾 |
| 175 | 冒险的 |
| 176 | 美好的 |
| 177 | 闷闷不乐 |
| 178 | 迷惑 |
| 179 | 迷恋的 |
| 180 | 迷人 |
| 181 | 迷失的 |
| 182 | 迷惘 |
| 183 | 勉强 |
| 184 | 藐视的 |
| 185 | 名誉扫地 |
| 186 | 漠不关心 |
| 187 | 漠然 |
| 188 | 目中无人 |
| 189 | 呆滞 |
| 190 | 耐心的 |
| 191 | 难过 |
| 192 | 难以置信 |
| 193 | 恼火 |
| 194 | 恼怒的 |
| 195 | 内疚的 |
| 196 | 逆反的 |
| 197 | 凝重 |
| 198 | 忸怩 |
| 199 | 虐待的 |
| 200 | 惊慌失措 |
| 201 | 品头论足 |
| 202 | 平淡无奇 |
| 203 | 平静 |
| 204 | 评判的 |
| No. | Word |
| 205 | 破灭的 |
| 206 | 期待的 |
| 207 | 欺骗的 |
| 208 | 乞求的 |
| 209 | 气恼的 |
| 210 | 残忍的 |
| 211 | 气馁的 |
| 212 | 谦虚 |
| 213 | 谦逊 |
| 214 | 谴责的 |
| 215 | 强横 |
| 216 | 钦佩的 |
| 217 | 亲密的 |
| 218 | 亲昵 |
| 219 | 轻蔑的 |
| 220 | 倾听的 |
| 221 | 请求的 |
| 222 | 屈从的 |
| 223 | 全神贯注 |
| 224 | 雀跃的 |
| 225 | 确信的 |
| 226 | 热爱 |
| 227 | 热情 |
| 228 | 痛苦 |
| 229 | 如坐针毡 |
| 230 | 撒谎的 |
| 231 | 煽动的 |
| 232 | 伤感的 |
| 233 | 诚实 |
| 234 | 伤怀的 |
| 235 | 深情 |
| 236 | 神经质的 |
| 237 | 神秘 |
| 238 | 神清气爽的 |
| 239 | 生气的 |
| 240 | 盛气凌人 |
| 241 | 失望的 |
| 242 | 受创的 |
| 243 | 受欢迎的 |
| 244 | 受回报的 |
| 245 | 受接纳的 |
| 246 | 受恐吓的 |
| No. | Word |
| 247 | 受虐的 |
| 248 | 受骗的 |
| 249 | 受欺负的 |
| 250 | 受挑逗的 |
| 251 | 受压迫的 |
| 252 | 受折磨的 |
| 253 | 舒适 |
| 254 | 思乡的 |
| 255 | 算计的 |
| 256 | 随和的 |
| 257 | 随意的 |
| 258 | 踏实 |
| 259 | 贪婪的 |
| 260 | 忐忑不安 |
| 261 | 陶醉的 |
| 262 | 讨人嫌的 |
| 263 | 讨厌的 |
| 264 | 挑拣的 |
| 265 | 挑衅的 |
| 266 | 通情达理 |
| 267 | 同感的 |
| 268 | 痛不欲生 |
| 269 | 热情 |
| 270 | 痛苦 |
| 271 | 痛心 |
| 272 | 投入的 |
| 273 | 威胁的 |
| 274 | 未雨绸缪 |
| 275 | 反感的 |
| 276 | 温馨 |
| 277 | 无动于衷 |
| 278 | 无精打采 |
| 279 | 被怠慢的 |
| 280 | 无礼的 |
| 281 | 无聊的 |
| 282 | 无奈的 |
| 283 | 无牵无挂 |
| 284 | 无情 |
| 285 | 无所适从 |
| 286 | 无忧无虑 |
| 287 | 希望的 |
| 288 | 喜爱的 |
| No. | Word |
| 289 | 喜欢的 |
| 290 | 戏弄的 |
| 291 | 嫌恶的 |
| 292 | 享受的 |
| 293 | 消沉 |
| 294 | 消极的 |
| 295 | 嚣张 |
| 296 | 小心翼翼 |
| 297 | 歇斯底里 |
| 298 | 胁迫的 |
| 299 | 搅扰的 |
| 300 | 泄气的 |
| 301 | 心不在焉 |
| 302 | 心烦意乱 |
| 303 | 心甘情愿 |
| 304 | 心灰意冷 |
| 305 | 心乱如麻 |
| 306 | 心碎 |
| 307 | 欣赏的 |
| 308 | 欣慰 |
| 309 | 欣喜若狂 |
| 310 | 信服的 |
| 311 | 信任的 |
| 312 | 兴奋的 |
| 313 | 兴高采烈 |
| 314 | 幸运 |
| 315 | 幸灾乐祸 |
| 316 | 羞耻 |
| 317 | 惊吓的 |
| 318 | 羞愧 |
| 319 | 羞辱的 |
| 320 | 虚伪 |
| 321 | 悬而未决 |
| 322 | 严厉 |
| 323 | 严肃 |
| 324 | 言听计从 |
| 325 | 厌恶的 |
| 326 | 厌烦 |
| 327 | 厌倦的 |
| 328 | 一脸凶相的 |
| 329 | 一意孤行 |
| 330 | 疑惑的 |
| No. | Word |
| 331 | 疑心的 |
| 332 | 隐瞒的 |
| 333 | 迎合的 |
| 334 | 忧愁 |
| 335 | 忧心忡忡 |
| 336 | 忧心的 |
| 337 | 幽默 |
| 338 | 犹豫 |
| 339 | 友好 |
| 340 | 友善的 |
| 341 | 鬼鬼祟祟 |
| 342 | 有活力的 |
| 343 | 有礼貌的 |
| 344 | 有魅力 |
| 345 | 有用的 |
| 346 | 诱惑的 |
| 347 | 诱人的 |
| 348 | 愉快 |
| 349 | 愉悦 |
| 350 | 愚蠢 |
| 351 | 郁闷 |
| 352 | 原谅的 |
| 353 | 赞赏的 |
| 354 | 责怪的 |
| 355 | 憎恶的 |
| 356 | 憎恨的 |
| 357 | 沾沾自喜 |
| 358 | 折服的 |
| 359 | 着迷的 |
| 360 | 对质的 |
| 361 | 振奋的 |
| 362 | 震撼的 |
| 363 | 震惊的 |
| 364 | 震怒的 |
| 365 | 镇静 |
| 366 | 争强好胜的 |
| 367 | 执意 |
| 368 | 趾高气昂 |
| 369 | 质疑的 |
| 370 | 挚爱的 |
| 371 | 置身事外 |
| 372 | 专横 |
| 373 | 专注的 |
| 374 | 自卑 |

| No. | Word |
| --- | --- |
| 375 | 自嘲的 |
| 376 | 自豪的 |
| 377 | 自鸣得意 |
| 378 | 感兴趣的 |
| 379 | 自私 |
| 380 | 自以为是 |
| 381 | 自责的 |
| 382 | 尊重的 |
